# Reverse Triggering: An Introduction to Diagnosis, Management, and Pharmacologic Implications

**DOI:** 10.3389/fphar.2022.879011

**Published:** 2022-06-22

**Authors:** Brian Murray, Andrea Sikora, Jason R. Mock, Thomas Devlin, Kelli Keats, Rebecca Powell, Thomas Bice

**Affiliations:** ^1^ University of North Carolina Hospitals, Chapel Hill, NC, United States; ^2^ College of Pharmacy, University of Georgia, Athens, GA, United States; ^3^ Augusta University Medical Center, Augusta, GA, United States; ^4^ Novant Health, Winston-Salem, NC, United States

**Keywords:** critical care, mechanical ventilation, reverse triggering, respiratory failure, acute respiratory distress syndrome, sedation

## Abstract

Reverse triggering is an underdiagnosed form of patient-ventilator asynchrony in which a passive ventilator-delivered breath triggers a neural response resulting in involuntary patient effort and diaphragmatic contraction. Reverse triggering may significantly impact patient outcomes, and the unique physiology underscores critical potential implications for drug-device-patient interactions. The purpose of this review is to summarize what is known of reverse triggering and its pharmacotherapeutic consequences, with a particular focus on describing reported cases, physiology, historical context, epidemiology, and management. The PubMed database was searched for publications that reported patients presenting with reverse triggering. The current body of evidence suggests that deep sedation may predispose patients to episodes of reverse triggering; as such, providers may consider decreasing sedation or modifying ventilator settings in patients exhibiting ventilator asynchrony as an initial measure. Increased clinician awareness and research focus are necessary to understand appropriate management of reverse triggering and its association with patient outcomes.

## 1 Introduction

**FIGURE 1:**
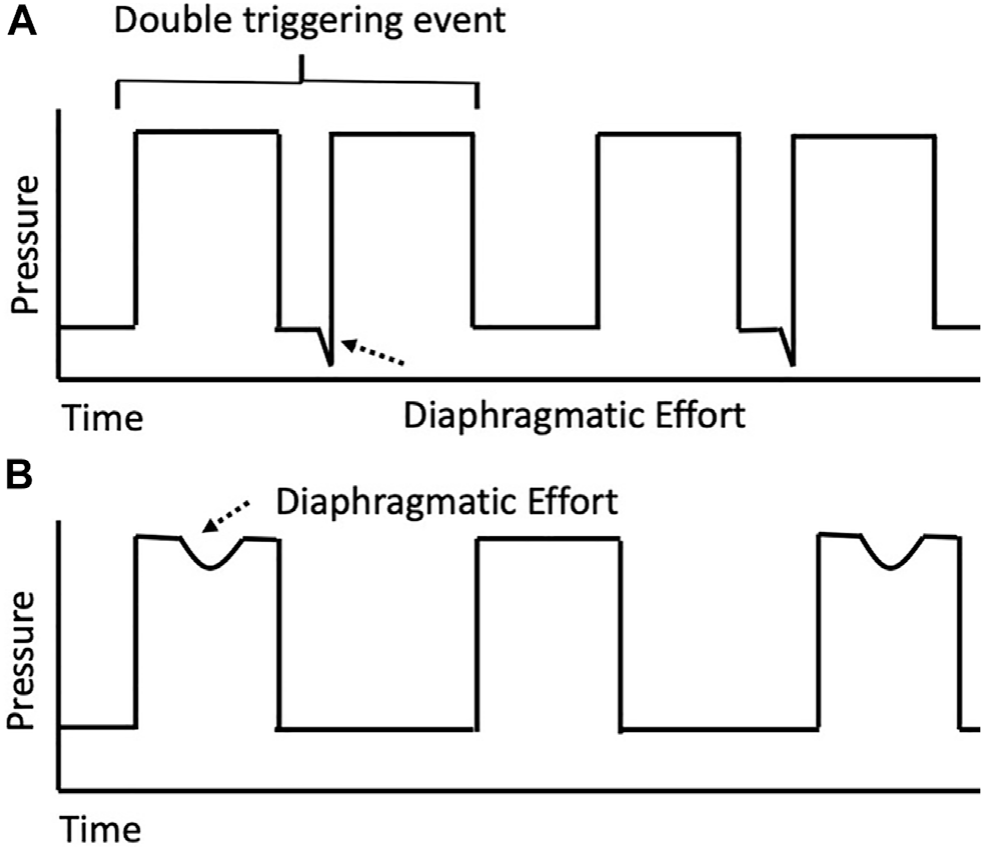
Simplified pressure-time curve for possible reverse triggering event. This simplified schematic provides an overview of a reverse triggering event. **(A)** Passive ventilator breaths followed by a discordant diaphragmatic effort during expiration in a 1:1 entrainment ratio. Such diaphragmatic efforts, if initiated in or persisting beyond the ventilator refractory period and significant enough to exceed the set trigger, will result in double-triggering as shown. **(B)** Passive ventilator breath with a diaphragmatic effort during the inspiratory phase in a 1:2 entrainment ratio. In this example, the diaphragmatic effort during the ventilator refractory period results in an apparent early or ineffective trigger.

**FIGURE 2:**
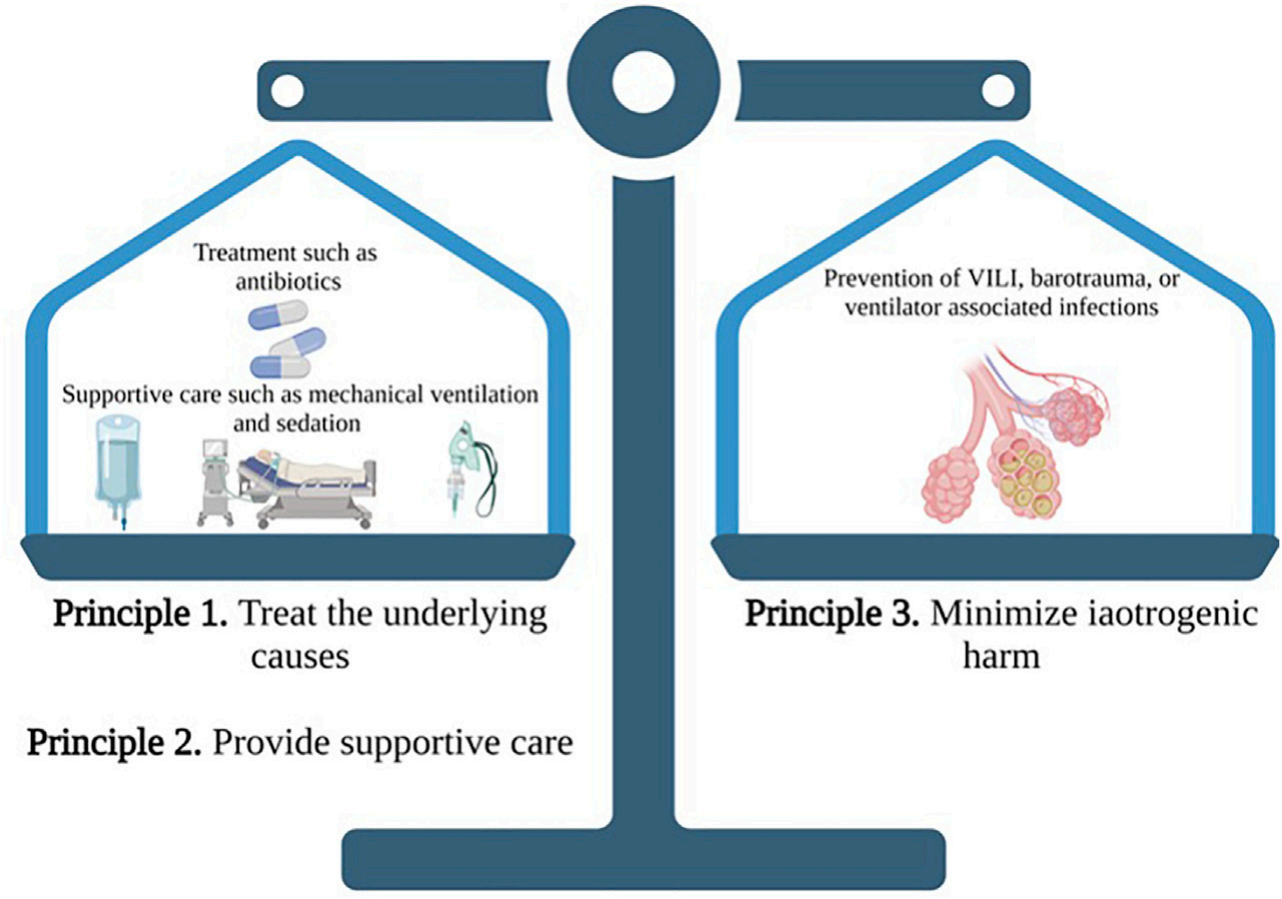
The three principles of critical care: A proposed schematic of balancing risks and harms of various interventions. Two special objects (must be kept) in view with regard to disease, namely, to do good or to do no harm—Hippocrates. With the three principles of critical care, the two principles of treatment of the underlying cause and provision of supportive care must be counterbalanced by the third principle of minimizing iatrogenic harm. When applied to a mechanically ventilated patient, treating the underlying cause may include pharmacotherapy (e.g., antibiotics) and non-pharmacotherapy (e.g., chest tubes); supportive care may be providing adequate oxygenation and ventilation while the therapies reverse the underlying cause (e.g., invasive positive pressure ventilation); and minimizing harm may include preventing VILI or ventilator associated infections. In the case of reverse triggering, two supportive care modalities (mechanical ventilation and sedation) may increase risk of iatrogenic harm, and careful consideration is warranted to maintain appropriate balance. *Source* Hanna Azimi, PharmD. Created with Biorender.com.

Reverse triggering is a unique form of patient-ventilator asynchrony that presents an important intersection of drug and device. Reverse triggering is a form of patient-ventilator asynchrony during which a passive ventilator-delivered breath triggers a neural response that results in an involuntary patient effort and diaphragmatic contraction. Figure 1 provides a basic schematic for this unique phenomenon. Globally, patient-ventilator asynchrony during invasive mechanical ventilation has been postulated to have important ramifications for patient outcomes (Akoumianaki et al., 2013). Mechanical ventilation, in a best-case scenario, is a supportive care strategy that “rests” the respiratory system and assists with gas exchange while allowing time to manage the underlying cause of respiratory failure; however, it may also cause direct patient harm and increase mortality through ventilator-induced lung injury (VILI) (Acute Respiratory Distress Syndrome et al., 2000; Antonogiannaki et al., 2017; Thille et al., 2006; Slutsky and Ranieri, 2013; Amato et al., 2015; Blanch et al., 2015). Careful management of this supportive care modality that minimizes iatrogenic harm poses a unique clinical challenge in the construct of balancing benefits and harms. While some aspects of VILI (i.e., use of injurious tidal volumes or pressures) is well supported, our understanding of the impact of spontaneous patient efforts (including ventilator asynchronies), more specifically categorized as patient self-inflicted lung injury (P-SILI), on patient outcomes continues to evolve (Yoshida et al., 2013; Yoshida et al., 2020; Carteaux et al., 2021). As such, ventilator asynchronies like reverse triggering may represent a modifiable risk factor for poor outcomes (de Haro et al., 2019a; Marini et al., 2020). Currently, no recommendations exist for managing patients that experience reverse triggering and limited evidence describes the impact of pharmacotherapeutic decisions on outcomes. This review of reverse triggering describes relevant pathophysiology, influence on patient outcomes, and interplay of pharmacotherapeutic decisions on ventilator outcomes. The discussion of reverse triggering is framed around balancing risks and harms associated with critical care interventions (Figure 2).

## 2 Methodology

A literature search was performed to identify studies including patients undergoing mechanical ventilation with reverse triggering events. The PubMed database was searched for English-language reports published between January 1995 and August 2021 using combinations of the search terms acute respiratory failure, acute respiratory distress syndrome, invasive positive pressure ventilation, mechanical ventilation, neuromuscular blockade, reverse triggering, sedation, ventilator-induced lung injury, and ventilator asynchrony. Table 1 reviews terminology associated with reverse triggering discussed in this review. Publications of all study designs that reported patients presenting with reverse triggering were included. Further, references found within original research articles, review articles, editorials, abstracts, meta-analyses, and systematic reviews were screened for inclusion.

**TABLE 1:** Relevant terminology.

Term	Definition
Reverse triggering	Diaphragmatic muscle contraction induced by passive insufflation of the lungs, especially in deeply sedated patients Akoumianaki et al. (2013), Yoshida et al. (2013)
Auto-triggering	When a cycle is delivered by the ventilator in absence of true patient effort Thille et al. (2006)
Breath-stacking	When the patient-triggered breath occurs at the end of passive inflation, and therefore increases the tidal volume, also known as double insufflation Akoumianaki et al. (2013)
Ventilator asynchrony	Occurs when either the mechanical breath is not in agreement with the neural inspiration of the patient, or if the extent of mechanical assist does not meet the patient’s respiratory demand Fan et al. (2017)
Respiratory entrainment	Repetitive relationship between the mechanical and neural respiratory cycles in which the patient-driven inspirations occur at a specific and repetitive rate Akoumianaki et al. (2013)

### 2.1 History of Reverse Triggering

**FIGURE 3:**
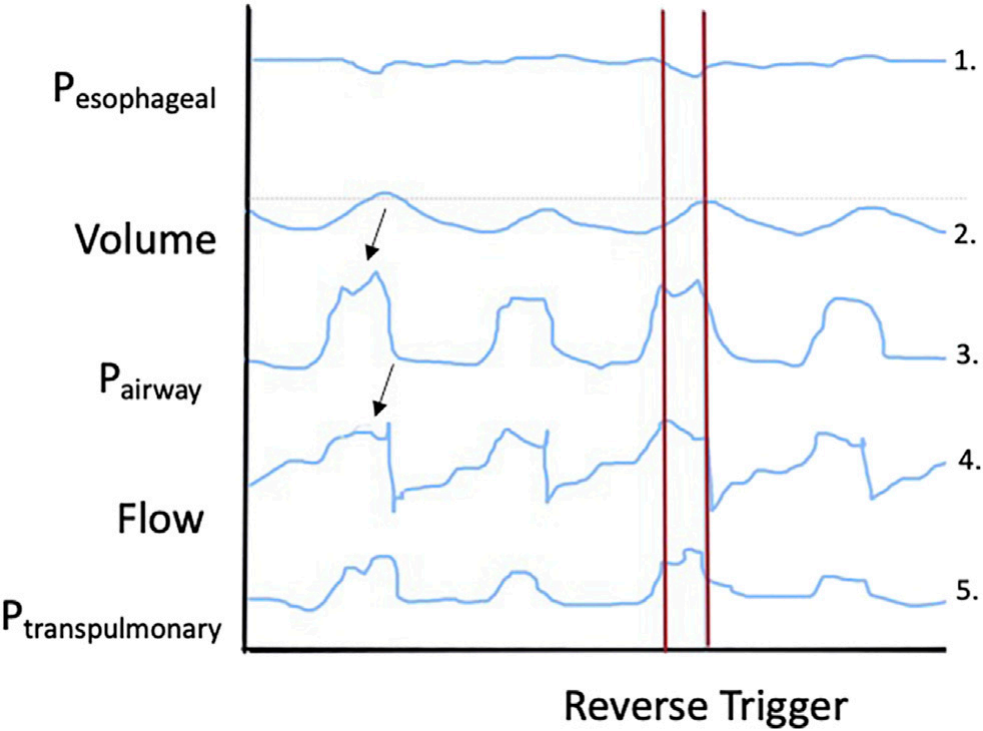
Theoretical depiction of a waveform tracing for a reverse triggering event. Key elements of this waveform tracing to note include: 1) a maximal patient effort occurring during lung inflation, 2) a larger tidal volume during the reverse trigger event, 3) inspiratory effort resulting in a slight negative dip in airway pressure, 4) sharply increased flow during the reverse trigger event, and increased transpulmonary pressures (both mean and end-inspiratory) during the reverse triggering event.

Reverse triggering was first described in 2013 by Akoumianaki et al. (2013) as a new form of ventilator asynchrony. Eight patients ventilated with either volume assist-control or pressure assist-control ventilation methods had respiratory mechanics monitored with an esophageal balloon catheter. The authors noted that the passive insufflation from the ventilator elicited a reflex neural response from the patient resulting in an additional diaphragmatic contraction. This additional patient effort, termed reverse triggering, could result in several types of ventilator asynchrony depending on the timing and magnitude of the effort (e.g., ineffective patient efforts and double triggering). Figure 3 depicts a potential waveform tracing for a reverse triggering event. This phenomenon has now been observed in additional patient cases and specifically in more deeply sedated patients (Supplementary Appendix Table A1) (Akoumianaki et al., 2013; Murias et al., 2016). Reverse triggering received more attention following the publication of the Reevaluation of Systemic Early Neuromuscular Blockade (ROSE) trial, which, in contrast to the 2010 ARDS et Curarisation Systematique (ACURASYS) trial, found no difference in 90-day mortality with the use of neuromuscular blocking agents (NMBA) for management of acute respiratory distress syndrome (ARDS) (Papazian et al., 2010; National Heart et al., 2019; Park and Schmidt, 2019; Slutsky and Villar, 2019). Subsequent expert opinion, seeking to provide potential answers for these conflicting results, implicated reverse triggering as a potential contributor to mortality due to its previous association with deep sedation and the notable difference in sedation practices in the control groups of ACURASYS and ROSE (Rubenfeld et al., 2005; Bellani et al., 2016). Despite the increased awareness, the implications and management of reverse triggering have only become more complicated in the decade after its first being reported.

### 2.2 Epidemiology of Ventilator Asynchrony and Reverse Triggering

Of the more than five million patients in the United States admitted to an ICU every year, up to 40% will be mechanically ventilated, with numbers substantially higher in the COVID-19 era (Rubenfeld et al., 2005; Bellani et al., 2016; Gupta et al., 2020). Bedside recognition of ventilator asynchrony is notoriously difficult even for experienced specialists, with the problem compounded by the fact that clinicians cannot directly observe every ventilator cycle, which can lead to sampling errors (Colombo et al., 2011). However, over 90% of mechanically ventilated patients are thought to experience ventilator asynchrony of some kind, with each type characterized by unique pathophysiology, risk factors, and incidence (Thille et al., 2006; Blanch et al., 2015). Like other forms of ventilator asynchrony, reverse triggering is underdiagnosed owing to the subtlety of the associated waveform changes that are often hidden in the passive breath (Baedorf Kassis et al., 2021). Most studies reporting the prevalence of reverse triggering only evaluate minutes (up to an hour) of a patient’s full intubation period, further limiting the ability to understand the full scope of the issue. Moreover, the definition used for reverse triggering (or other asynchronies) influences the reported rates. As such, the true incidence of reverse triggering is unknown, but evidence suggests it is prevalent. One study found that 30% of patients with ARDS not receiving NMBA exhibited signs of reverse triggering, while another discovered evidence of reverse triggering in 50 of 100 patients (Bourenne et al., 2019; Rodriguez et al., 2020a). Most recently, multiple phenotypes have been proposed including early, mid-cycle, and late reverse triggering, which may all have unique incidences and causative factors (Baedorf Kassis et al., 2021).

### 2.3 Pathophysiology of Reverse Triggering

Reverse triggering occurs when passive ventilator insufflations trigger involuntary patient effort, seen as diaphragmatic muscle contractions [or, less frequently, respiratory accessory muscle contractions (Turbil et al., 2020)], which may or may not result in a subsequent breath being delivered by the ventilator (Akoumianaki et al., 2013; de Haro et al., 2019a). The physiological mechanism behind reverse triggering is not entirely understood. Still, a popular theory implicates the vagally mediated Hering-Breuer reflex, normally intended to protect the lungs from over-inflation by terminating inspiration in response to activation of mechanical stretch receptors (Baedorf Kassis et al., 2021). In reverse triggering, flow delivered by the ventilator is thought to activate stretch receptors in the upper airways, lung, and chest wall that provide afferent feedback to the respiratory center, which then matches the frequency of the external stimulus (Simon et al., 2000). However, reverse triggering has also been described in bilateral pulmonary transplant patients despite resection of vagal afferents, suggesting that vagal feedback may be sufficient but not necessary to elicit this phenomenon (Simon et al., 2000). Another report describes reverse triggering in two patients with brain death and loss of brainstem reflexes, suggesting that even the respiratory center may not be required (Delisle et al., 2016). These reports imply the involvement of other afferents (e.g., thoracic mechanoreceptors) or spinal reflexes (Delisle et al., 2016). Taken together, the physiology of reverse triggering appears more complex than once thought, and the possibility exists that one or multiple of these mechanisms may be involved in a critically ill patient.

The ventilator-induced neural responses seen in reverse triggering are thought to result from a phenomenon known as “entrainment” (Akoumianaki et al., 2013; Bourenne et al., 2019). Entrainment refers to the synchronization of phase (i.e., timing of a cycle) and period (i.e., duration of a cycle) of an oscillatory process to the rhythm of external input, as with electrical currents or brain waves. Respiratory entrainment, therefore, occurs when the external rhythm imposed by the ventilator is matched by the patient’s respiratory center (Akoumianaki et al., 2013; de Vries et al., 2019). Entrainment is known to occur in anesthetized animals undergoing mechanical ventilation, and in healthy human subjects in the waking, sleep, and anesthetized states (Simon et al., 1999). In non-pathologic states, entrainment may facilitate synchronization between the patient and the ventilator indicating the patient’s ability to modify breathing in response to the external stimulus. This response may reflect the involvement of the frontal cortex and higher-order control. At the same time, it has been postulated that a more deeply sedated state may unmask the respiratory system’s underlying reflexes. Entrainment seems to occur more frequently at respiratory rates or tidal volumes that are in line with normal spontaneous breathing, and levels of PCO_2_, which are known to affect respiratory drive, do not appear to influence the occurrence of entrainment (Simon et al., 1999).

In applying Akoumianaki et al.’s original definition, the entrainment ratio in reverse triggering remains phase-locked and stable (e.g., 1:1, 1:2, 1:3), with a 1:1 ratio (one patient effort for every passive machine-delivered breath) being most common. Periods of reverse triggering with a stable ratio may be interrupted by periods with no asynchrony, and the ratio may change over time (e.g., from 1:1 to 1:2). When entrainment occurs, the phase delay between the machine-triggered breath and the patient effort and the duration and magnitude of the patient effort remain constant (Akoumianaki et al., 2013; Murias et al., 2016; de Vries et al., 2019). However, whether a specific and stable ratio must be present to define an asynchrony event as reverse triggering remains controversial. Notably, several case series report reverse triggering with unstable ratios but acknowledge that these events may represent a complete uncoupling of the patient’s respiratory rhythm and the ventilator cycle highlighted by random spontaneous patient efforts as opposed to reverse triggering. Whether this nuanced distinction has any relevance to management or patient outcomes is unclear.

### 2.4 Consequences of Reverse Triggering

Expert opinion favors that the spontaneous effort associated with ventilator asynchronies can increase the transpulmonary pressure and distension, all thought to worsen lung injury and VILI-associated increases in the duration of mechanical ventilation, ICU length of stay, and mortality. However, the actual physiological effects and impact of reverse trigging on patient outcomes are unknown (Thille et al., 2006; Akoumianaki et al., 2013; Blanch et al., 2015; Murias et al., 2016; Mauri et al., 2017).

The most apparent mechanism of potential reverse triggering induced harm is *via* double triggering, or breath stacking. If the patient’s effort is strong enough and persists into exhalation, this reverse trigger may result in a second tidal volume breath being delivered by the ventilator, resulting in the patient receiving double the prescribed tidal volume. This stacked breath will then result in pulmonary over-distention and high trans-pulmonary pressures. A study examining the mechanisms of double triggering found that while the overall frequency was low and independent patient effort was the most common cause, reverse triggering was still implicated in over one-third of double triggering events (de Haro et al., 2018). In one cohort of ARDS patients receiving low tidal volume ventilation (LTVV), a high frequency of double triggering was reported; notably, this double triggering occurred despite the use of deep sedation intended to abolish spontaneous patient efforts (Pohlman et al., 2008).

Even in the absence of observed double triggering events or injurious tidal volumes, reverse triggering that causes ineffective respiratory muscle contraction within an inspiratory cycle has been shown to adversely affect pulmonary dynamics, leading to increased dependent lung stretch equivalent to what would be expected with a 15 ml/kg tidal volume (Yoshida et al., 2018). This inflation pattern can be explained by the Pendelluft effect (literally “swinging air”) (Greenblatt et al., 2014). In this phenomenon, non-homogeneous inflation and deflation creates regional pressure differences in the lung and airflow among different lung regions, thus increasing regional tidal volumes and transpulmonary pressures (Akoumianaki et al., 2013; Bourenne et al., 2019). The negative intrathoracic pressures associated with reverse-triggered breaths can also predispose to alveolar edema (de Vries et al., 2019).

Reverse triggering is also thought to directly affect the diaphragm, though whether this ultimately results in injury or protection is controversial. Diaphragmatic contractions *via* reverse triggering in a patient that is otherwise completely passively ventilated may help to preserve some muscle activity and strength, (Hooijman et al., 2015; van den Berg et al., 2017) which may ultimately shorten time on the ventilator. Conversely, eccentric contraction of the diaphragm during exhalation (as can occur with reverse triggering) may cause diaphragmatic muscle fiber damage (Goligher et al., 2015). Regardless of the net effect, patient effort related to reverse triggering may increase work of breathing, oxygen consumption, and CO_2_ production (Chanques et al., 2013; He et al., 2018).

### 2.5 Recognition of Reverse Triggering

Limited data are available for the recognition of reverse triggering. However, experienced clinicians may be able to use simple bedside maneuvers and careful monitoring of the patient and ventilator waveforms to distinguish reverse triggering from other forms of ventilator asynchrony. To evaluate possible reverse triggering, the mandatory ventilator rate may be reduced (potentially as low as 8–10 breaths per minute) to allow evaluation of the patient’s underlying spontaneous efforts. For apparent double-triggering type reverse trigger: if the patient continues to breathe at a fast rate, and double triggering still occurs, then reverse triggering is not the culprit for the asynchrony. Either premature-cycling or flow starvation is potentially the cause. If the patient’s ventilatory rate drops and there are only spontaneous efforts following mandatory ventilator-delivered breaths, then the double triggering is almost certainly due to reverse triggering. If the patient continues to make spontaneous efforts and the trigger disappears, then this is more likely a mismatch of vent-rate and patient set rate, which is likely due to over-sedation and is another type of reverse trigger. If the patient’s respiratory rate drops and there is an apparent trigger only during mandatory ventilator-delivered breaths, then this is highly suspicious for reverse triggering.

While bedside monitoring and maneuvers may be simple to perform, they require that clinicians must first be present at the time of the asynchrony to evaluate and must also be familiar with the classic signs that merit further workup of reverse triggering. Techniques and tools such as esophageal pressure monitoring or electromyography monitoring of the diaphragm in addition to new software and machine learning algorithms have shown promise and allow for the capture of even subtle signals on a continuous basis (Rodriguez et al., 2020b; Pham et al., 2021).

### 2.6 Pharmacologic Considerations

**FIGURE 4:**
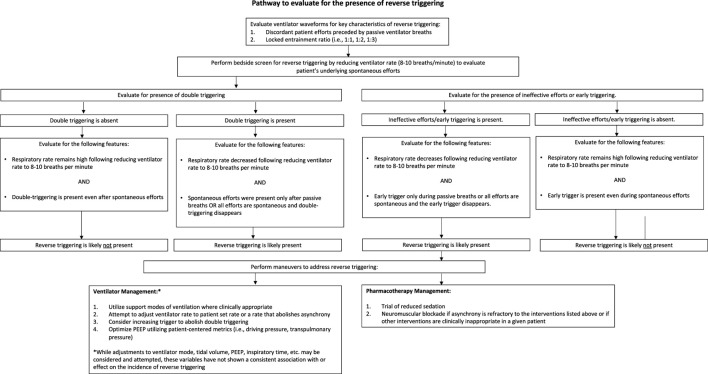
Proposed approach to the patient with ventilator asynchrony and possible reverse triggering.

Beyond direct physiologic effects of reverse triggering, potential consequences of inappropriate or aggressive medical management of this unique patient-ventilator synchrony are a concern. Ventilator asynchrony has been historically managed by fine-tuning ventilator settings based on patient-ventilator interactions and pharmacologic interventions including analgesia, sedation, and NMBA (Chanques et al., 2013; Goligher et al., 2015; He et al., 2018). However, these interventions may not be optimal for patients exhibiting reverse triggering as their mechanism of ventilator asynchrony (National Heart et al., 2019; Park and Schmidt, 2019; de Vries et al., 2019; de Wit et al., 2009). Indeed, attempting to abort the asynchrony with deeper levels of sedation, while also potentially being ineffective, will undoubtedly lead to longer durations of mechanical ventilation and associated complications (Kress et al., 2000; Treggiari et al., 2009). Additionally, the use of NMBA, while effective at terminating reverse triggering events, may be employed when less aggressive interventions would have sufficed. Figure 4 proposes a potential pathway to reverse triggering management based on assessment of the available literature; however, it is notable that no formalized reverse triggering treatment approach or protocol has been robustly evaluated. The authors caveat all suggestions with this in mind and consider this a key area for future research and delineation.

#### 2.6.1 Analgesia and Sedation

Increasing the depth of sedation with analgesics and sedatives is a management strategy for patient-ventilator asynchrony. It effectively reduces the incidence of some forms of asynchrony by suppressing the patient’s respiratory drive (Bassuoni et al., 2012; Sottile et al., 2018). Sottile et al. (2018) found that deep sedation (i.e., RASS −4 to −5) resulted in a significantly lower incidence of all types of ventilator asynchrony, including double triggering, when compared to light sedation (i.e., RASS 0 to −1). However, deeper levels of sedation have been associated with a higher incidence of ineffective patient efforts in several studies (Kress et al., 2000; Sottile et al., 2018). This effect is related to decreased inspiratory muscle effort and a lower maximal inspiratory flow with increased sedative dosage (de Wit et al., 2009).

Counter-intuitive to traditional clinical reasoning, reverse triggering incidence may increase with depth of sedation and has been reported during assist control ventilation under deep sedation in multiple studies (Akoumianaki et al., 2013; Chanques et al., 2013; He et al., 2018). One case of reverse triggering in a patient with ARDS reported resolution of asynchrony when sedation was discontinued and the patient regained consciousness (Ueno et al., 2017). However, recent literature has been unable to show a significant correlation between sedative dosing and incidence of reverse triggering (Rodriguez et al., 2020a). Other reports suggest that the occurrence of reverse triggering may represent the transition between deep sedation and recovery of the patient’s respiratory drive (Mellado Artigas et al., 2021).

In sum, deep sedation may abolish behavioral responses or stronger cortical influences that affect ventilator interactions in the awake state, potentially promoting reverse triggering (Akoumianaki et al., 2013). Given the relatively recent description of reverse triggering as a unique form of patient-ventilator asynchrony, minimal literature is available on the relationship between sedation levels or sedative choice and reverse triggering, and national guidelines provide minimal direction regarding how opioids and sedatives should be optimally employed for patient-ventilator asynchrony (Devlin et al., 2018; Yoshida et al., 2018; Murray et al., 2016; Fan et al., 2017; Rhoney and Murry, 2003). Globally, the relationship between asynchronies of all kinds (including reverse triggering), sedation level, and sedative and opioid agent are minimally characterized. Such nuances of dosing strategy (e.g., route, duration, cumulative dose, etc.) have not been evaluated. Although one study showed potentially improved asynchrony index with dexmedetomidine versus propofol, opioids were not taken into account (Conti et al., 2016). Some studies have shown improved asynchrony index through the use of opioids but did not report evaluation of reverse triggering, specifically (Richman et al., 2006; de Haro et al., 2019b). Future focus on the interaction of agent, dosing strategy, device, and patient are warranted. Notably, despite some conflicting reports, expert opinion favors deep sedation as a risk factor for reverse triggering, and higher quality case series of reverse triggering tend to corroborate this relationship. Overall, a trial of decreased sedation and analgesia as a potential means to combat reverse triggering should be attempted before more aggressive measures are taken.

#### 2.6.2 Neuromuscular Blocking Agents

The confounding role of reverse triggering in trials of NMBA has implications not only for understanding the role of NMBA in ARDS but also for the management of reverse triggering in the clinical setting. In the setting of mechanical ventilation, NMBA are generally reserved for cases of refractory hypoxemia due to ARDS but may exert their clinical benefit primarily by aborting and/or preventing ventilator asynchrony (including reverse triggering) that can lead to VILI. One of the strongest arguments in favor of the clinical significance of reverse triggering and the impact of deep sedation on its incidence comes from Park and Slutsky. They proposed that the difference in outcomes between the ACURASYS and ROSE trials was due to differences in sedation goals in the control arms of each study. While ROSE targeted light sedation in the control group in accordance with current best practice, ACURASYS targeted deep sedation in both trial arms (Papazian et al., 2010; National Heart et al., 2019; Park and Schmidt, 2019; Slutsky and Villar, 2019). Thus, patients in the ACURASYS control group were more deeply sedated (although doses were not recorded), which would potentially lead to a higher incidence of reverse triggering (Papazian et al., 2010; Park and Schmidt, 2019). While episodes of patient-ventilator asynchrony were not measured in either trial, an increased incidence of reverse triggering in the ACURASYS control group could have increased rates of VILI, leading to worse outcomes in patients not receiving NMBA (Park and Schmidt, 2019). Interestingly, a significantly higher number of patients developed a pneumothorax in the control group than those who received NMBA, which could be consistent with an increased incidence of ventilator asynchrony (Papazian et al., 2010). However, because neither trial reported incidence of ventilator asynchrony, indices of transpulmonary pressure, or levels of inflammatory cytokines, the contribution of asynchronies (and prevention of asynchronies by NMBA) to patient survival and ventilator-free days is purely speculative (Park and Schmidt, 2019).

NMBA may be beneficial in patients experiencing ventilator asynchrony (e.g., breath stacking) and in those who have high transpulmonary pressure swings, and they are effective for aborting reverse triggering (Slutsky and Villar, 2019). For patients experiencing episodes of reverse-triggering, it may be reasonable to consider a trial of light sedation before using NMBA. Still, the addition of NMBA may be considered if asynchrony is persistent despite changes to sedation levels and the ventilator. The benefits of NMBA must be weighed against their significant adverse effect profile, including exacerbation of diaphragmatic dysfunction during mechanical ventilation, which, when combined with the deeper levels of sedation required for these paralyzed patients, could also result in longer durations of mechanical ventilation (Testelmans et al., 2006).

### 2.7 Interventions Through Ventilator Settings

Optimizing ventilator settings is a central strategy to management of reverse triggering, from simply increasing patient comfort (i.e., “fitting the ventilator to the patient”) to potentially aborting the asynchrony without having to intensify pharmacotherapeutic interventions. While data regarding adjusting ventilator strategies to resolve reverse triggering are limited to retrospective studies and case reports, robust evidence for managing ventilator settings as an intervention for other types of asynchrony supports this avenue of intervention (Chanques et al., 2013). Notably, some studies have shown that interventions to the ventilator specifically targeting the type of asynchrony identified may prove more effective than making adjustments to sedation regimens (Chanques et al., 2013). Many case reports of reverse triggering describe patients receiving volume assist-control ventilation, a mode known to increase rates of asynchrony (Akoumianaki et al., 2013; He et al., 2018). However, reverse triggering has also been reported in patients receiving pressure-regulated or pressure-controlled modes of ventilation (Baedorf Kassis et al., 2021). While some patients may not be able to tolerate a change to a more comfortable mode such as pressure support ventilation (e.g., chest wall injuries), and some patients may not be able to be safely managed on pressure support ventilation (e.g., those that require low tidal volumes), changing to a support mode of ventilation may potentially abort reverse triggering and clinicians can consider changing ventilation modes if it can be done safely in patients that are repeatedly experiencing asynchrony (He et al., 2018).

Other changes to ventilator settings (e.g., ventilator rate, tidal volume, PEEP) may also abolish reverse triggering in certain patients. However, the relationship between any of these variables and the incidence of reverse triggering has not been clearly defined. Higher levels of PEEP have been postulated to cause more “stretch” in the respiratory system, potentially predisposing patients to reverse triggering; however, Yoshida et al. has proposed a higher PEEP strategy to reduce the injurious effects of high respiratory drive in severe lung injury (Morais et al., 2018). Here, higher PEEP is used to create a more even pressure distribution to reduce the risk of pendelluft based overdistension and generally higher PEEP levels are considered to improve gas exchange, which can reduce overall respiratory drive (Baedorf Kassis et al., 2021). Similarly, larger tidal volumes are also theorized to cause more “stretch” and therefore an increased likelihood of reverse triggering (de Vries et al., 2019); however, some observations suggest that lower tidal volumes are associated with a higher frequency of reverse triggering and increasing tidal volume has been shown to abolish reverse triggering (Akoumianaki et al., 2013; Rodriguez et al., 2020a). Alterations to respiratory rate may reduce reverse triggering as previously described; (Mellado Artigas et al., 2021) either increasing the ventilator rate or, more commonly, decreasing the ventilator rate until all breaths are initiated by patient effort, may be effective strategies for abolishing reverse triggering. For patients demonstrating double triggering associated with their reverse trigger, increasing the trigger threshold required to initiate a breath (i.e., setting a higher flow or a more negative pressure that the patient will need to generate to trigger another breath from the ventilator) may decrease the incidence of double triggering but will likely not resolve the reverse trigger (notably, too insensitive of a trigger also has adverse ramifications due to potentially creating high airway pressures); those events will instead be noted as ineffective triggers on the ventilator waveform. If reverse triggering events are refractory to ventilator changes or if ventilator changes cannot be attempted safely, altering sedation levels and/or trialing NMBA and evaluating their effects on asynchrony is likely warranted (Garofalo et al., 2018; Bruni et al., 2019). In sum, interventions proposed in Figure 4 must align with the three principles of critical care and will require future investigation for a definitive pathway.

## 3 Conclusion

The inherent complexity of patient-ventilator interactions, in addition to the heterogeneity of the studies that have evaluated these interactions, results in diverse and challenging clinical scenarios. In particular, reverse triggering requires increased clinician and researcher awareness to delineate its pathophysiology, prevalence, impact on patient outcomes, and optimal management strategies. Given the impact of ventilator-induced lung injury as well as sedation practices and NMBA use on duration of mechanical ventilation and morbidity, a protocolized approach to assessment for and management of reverse triggering would be expected to improve patient outcomes. Clinicians are advised to remember that, when providing supportive care with mechanical ventilation, medication and device are not siloed interventions but require thoughtful, interprofessional consideration to develop a management strategy that maximizes benefit and minimizes iatrogenic harm (Antonogiannaki et al., 2017).
