# Supplementary material for: Reverse Triggering: An Introduction to Diagnosis, Management, and Pharmacologic Implications
Source: Front Pharmacol. 2022 Jun 22;13:879011. doi: 10.3389/fphar.2022.879011 (PMC9256988; doi:10.3389/fphar.2022.879011)
Supplement: Supplementary file 1 [file Table1.docx]

**Appendix**

**Table 1. Summary of Reported Cases of Reverse Triggering**

| **Article** | **Patient** **Demographics** | **Diagnosis** | **Mechanical Ventilation Parameters** | **Sedation Methods** | **Method of Detection** | **Cause** | **Outcomes** |
| --- | --- | --- | --- | --- | --- | --- | --- |
| Artigas et al, 2021^49^ | Single-center study of 39 patients | Various medical reasons | *Median; greater than 8% incidence of RT (n=19) vs. less than 8% incidence (n=20)*  **Mode:** volume or pressure assist-control  **PEEP**: 8 vs. 9  **RR**: 20 vs 28  **Vt per predicted BW**: 6.8 vs 6.5 | 65% propofol  34% midazolam  Average Riker SAS: 2 | EAdi automatic detection of reverse triggering | Unknown | Unknown |
| Blokpoel et al, 2019^58^ | 11-month-old infant | Post-procedural subglottic edema-induced respiratory failure | **Mode**: time-cycled, pressure-limited  **Insp. pressure**: 20 cmH_2_O  **PEEP**: 5 cmH_2_O  **Inspiratory time**: unknown  **FiO2**: unknown  **RR**: 30  **Flow trigger**: 1.0 L/min | Propofol  Target COMFORT scale: 10 (high levels of sedation and pain control) | EAdi showed activity of the diaphragm between two machine breaths, indicative of reverse triggering | Unknown | Unknown |
| He et al, 2018^40^ | 55-year-old male | Chest trauma with multiple rib fractures | **Mode**: pressure assist-control  **Insp**. **pressure**: 15 cmH_2_O  **PEEP**: 8 cmH_2_O  **Inspiratory time**: 1s  **FiO_2_**: 0.4  **RR**: 20  **Flow trigger**:1.0 L/min  **Vt:** 430-460 mL | Midazolam and sufentanil continuous infusion  Target RASS: -4 to -5 (no response to voice but any movement to physical stimulation) | Ventilator screen showed mismatch between the flow and the airway pressure waveforms | Unknown | Reduced RR to 14 (below the patient’s spontaneous rate) and no reverse-triggered breath was identified |
| Ueno et al, 2017^48^ | 81-year-old male | ICU admission status post tricuspid valvuloplasty | **Mode**: pressure control  **Insp**. **pressure**: 10 cmH_2_O  **PEEP**: 6 cmH_2_O  **Inspiratory time**: 1.2s  **FiO_2_**: 0.5  **RR**: 12 | Fentanyl continuous infusion at 25 mg/h  RASS: -5 after 3 hours | EAdi monitoring revealed regular diaphragmatic contraction initiated about 1 second after start of mandatory breath resulting in double cycling. | Fentanyl, RASS -5 | Fentanyl was stopped, spontaneous breathing increased, and reverse triggering and double cycling disappeared |
| de Vries et al, 2019^29^ | 35-year-old male | Hypercapnic respiratory failure secondary to CAP | **Mode**: pressure control  **Insp**. **pressure**: 36 cmH_2_O  **PEEP**: 12 cmH_2_O  **Inspiratory time**: unknown  **FiO2**: 0.6  **RR**: 30  **Vt:** 502 mL  **Vt per IBW:** 5.7 mL/kg | Unknown | Ventilator screen was initially interpreted as “fighting the ventilator” which increasing doses of sedatives did not resolve | High inspiratory support levels | Rocuronium was administered to prevent reverse-triggered efforts after attempts to reduce sedation and alter ventilator parameters failed |
| Delisle et al, 2016 (case 1)^28^ | 40-year-old male | Brain death & massive cerebral edema status post out-of-hospital cardiac arrest and prolonged CPR | **Mode**: volume control  **Insp**. **pressure**: unknown  **PEEP**: 5 cmH_2_O  **Inspiratory time**: unknown  **FiO_2_**: unknown  **RR**: 20  **Vt:** 560 mL | Unknown | EAdi indicated patient–ventilator asynchrony suggesting “double triggering” | Unknown | Patient was placed on continuous positive airway pressure of 5 cm H_2_O and the asynchrony was replaced by apnea, thus confirming the absence of spontaneous breathing activity |
| Delisle et al, 2016 (case 2)^28^ | 78-year-old male | Out-of-hospital cardiac arrest complicating an acute upper airway obstruction | **Mode**: volume control  **Insp**. **pressure**: unknown  **PEEP**: 5 cmH_2_O  **Inspiratory time**: unknown  **FiO2**: unknown  **RR**: 10  **Vt:** 400 mL | None | Ventilator asynchrony was described as “regular and cyclic gasps” generating strong abdominal movements on 1:2 mechanical cycles | Unknown | No spontaneous respiratory activity was observed, withdrawal of life-sustaining therapies was performed on the following day |

PEEP = positive end-expiratory pressure, FiO2 = fraction of inspired oxygen, RR = respiratory rate (breaths per minute), EAdi = electrical activity of the diaphragm, Riker SAS = Riker Sedation Agitation Scale, RASS = Richmond Agitation-Sedation Scale, ETT = endotracheal tube, ICU = intensive care unit, CAP = community acquired pneumonia, CPR = cardiopulmonary resuscitation
